# Data-driven classification of narrative speech characteristics in stroke aphasia distinguishes neurological and strategic contributions

**DOI:** 10.1016/j.cortex.2025.03.006

**Published:** 2025-03-25

**Authors:** Junhua Ding, Daniel Mirman

**Affiliations:** a State Key Laboratory of Cognitive Science and Mental Health, Institute of Psychology, Chinese Academy of Sciences, Beijing, China; b Department of Psychology, University of Edinburgh, Edinburgh, UK

**Keywords:** Narrative speech, Quantitative production analysis, Correct information unit, Communication strategy, Stroke aphasia

## Abstract

Narrative speech deficits are common in post-stroke aphasia, resulting in negative influences on social participation and quality of life. Speech rate, complexity, and informativeness deficits all contribute to narrative speech. Research studies typically (implicitly) assume that these aspects of narrative speech production are a result of cognitive/neurological impairment, but they may also result from strategic choices made as individuals with aphasia attempt to produce narrative speech. Here, we used data-driven methods to classify aphasic narrative speech patterns and evaluated their predictability from lesion patterns. 76 stroke aphasia patients completed 11 narrative speech production tasks. Quantitative Production Analysis (QPA) and Correct Information Unit (CIU) analysis were used to measure their structural and functional properties. Based on prior work, we selected QPA measures of speech rate (words per minute) and complexity (mean sentence length, inflection index, and auxiliary index) and four CIU measures of informativeness (#CIU, CIU/min, %CIU, #nonCIU). These measures produced two orthogonal dimensions with four orthogonal participant clusters. Comprehensive comparison between clusters revealed that speech rate and complexity were strongly associated with general aphasia severity and total lesion volume, and were predicted by frontoparietal grey matter and dorsal pathway white matter damage. In contrast, informativeness was independent of other behavioral and neurological deficits, and was not predictable from lesion patterns, suggesting that it reflects communication strategy rather than specific neurological impairment. These results provide an important step toward distinguishing neurological and strategic aspects of narrative speech deficits in post-stroke aphasia, with potential implications for treatment approaches that target communication strategies.

## 1. Introduction

Spoken language is the most common form of human communication, but this ability is impaired for 40%–60% stroke survivors (Flowers et al., 2016), resulting in severe negative impacts on social participation and quality of life (Lee, Lee, Choi, & Pyun, 2015; Pike, Kritzinger, & Pillay, 2017). However, speech production is a complex process (Garrett, 1980; Levelt, Roelofs, & Meyer, 1999), requiring rapid coordination of articulatory, phonological, semantic, syntactic, and discourse processes. Reflecting this complexity, contemporary aphasia classification is based on multidimensional approaches (Ingram et al., 2020; Landrigan, Zhang, & Mirman, 2021).

Narrative, connected speech can be described in terms of its rate (e.g., words per minute), its structural complexity (syntax), its lexical-semantic diversity (e.g., number of unique words), and its informativeness or communicative success (Armstrong, 2000; Pritchard, Hilari, Cocks, & Dipper, 2017). Quantitative Production Analysis (QPA) was developed to measure structural impairments of speech, irrespective of the contents (Saffran, Berndt, & Schwartz, 1989). QPA uses measures such as mean sentence length and proportion of words that are in sentences to capture the syntactic well-formedness and complexity of narrative speech (Berndt, Schwarz, & Saffran, 2001; Gordon, 2006). Recent lesion-symptom mapping (LSM) analyses have found that sentence complexity and structural accuracy are associated with frontoparietal damage and, in the chronic phase, with overall lesion size (Ding, Martin, Hamilton, & Schnur, 2020; Mirman, Kraft, Harvey, Brecher, & Schwartz, 2019; Zevgolatakou, Thye, & Mirman, 2023).

In contrast to the structural focus of QPA, Correct Information Unit (CIU) analysis is designed to measure the informativeness and efficiency of narrative speech from a functional perspective (Nicholas & Brookshire, 1993). A correct information unit (CIU) is counted if it is relevant to the topic, regardless of the syntactic complexity or accuracy of the utterance where it occurs. As a result, it captures functional aspects of narrative speech independent of structural properties. Damage to frontoparietal white matter tracts is associated with reduced quantity and speed of CIU production (Ding, Middleton, & Mirman, 2023; Marchina et al., 2011).

Although both QPA and CIU are widely-used approaches, there are still few studies trying to combine these two systems in an aphasia cohort. The Linguistic Underpinnings of Narrative in Aphasia (LUNA) framework provides a way to unify the pragmatic, macrostructural, propositional and linguistic aspects (Dipper et al., 2021). Studies within this framework have begun to investigate relationships between main concepts, informativeness (CIU measures), and grammatical structure (Kuvač Kraljević, Matić Škorić, & Lice, 2023). Alyahya et al. (2022) explored the relationship between some microstructural (words/min and number of words) and macrostructural (word accuracy) discourse measures, and their lesion correlates. Their results showed a dissociation between these two types of measures, so-called ‘verbal quantity’ and ‘quality’ and dissociated neural correlates. Combining QPA and CIU measures in a single study would build on this prior work, with QPA providing further measures of syntactic complexity and CIU adding informativeness measures such as the number and rate of correct words. Whether the QPA and CIU are also independent is unknown. Lesion mapping studies suggest distinct lesion correlates: QPA scores are associated with frontoparietal grey matter damage, while CIU scores are associated with dorsal white matter integrity (Ding et al., 2020, 2023; Marchina et al., 2011; Zevgolatakou et al., 2023). However, individuals with aphasia may adapt their speech patterns to achieve better functional communication. For example, non-fluent/agrammatic individuals may shorten their sentences but use more informative words (Fedorenko, Ryskin, & Gibson, 2023; Rezaii, Michaelov, et al., 2023); individuals with semantic or word retrieval difficulties may use circumlocution strategies (Sajjadi, Patterson, Tomek, & Nestor, 2012). Lesion analysis may help to identify deficit dimensions that correspond to strategic choices as well as those that result from impaired neural systems.

In the current study, we first combined CIU and QPA measures derived from narrative speech to examine relationships between speech rate, complexity, and informativeness in a relatively large, unselected sample of participants with post-stroke aphasia. We characterize how these measures could generate two orthogonal components (i.e., QPA and CIU) and define distinct clusters of participants along the two components. Second, we used grey and white matter damage to distinguish the clusters. If cluster membership can be predicted based on lesion patterns, those differences have a neuroanatomical basis. If cluster membership *cannot* be predicted from lesion patterns, that suggests that the differences may be due to strategic choices rather than impaired neural systems.

## 2. Material and methods

### 2.1. Participants

This study combined data from two of our previous studies (Ding et al., 2023; Zevgolatakou et al., 2023): 76 participants who completed speech production tasks for both CIU and QPA analyses. Table 1 shows the demographic and neuropsychological information for the participants. All participants were in the chronic phase following left-hemisphere stroke, were native English speakers, right-handed, had no other brain disorders (e.g., dementia, tumor, encephalitis, epilepsy, psychosis, blindness/deafness), and were able to produce some intelligible speech (at least one correct response on a picture naming test). All participants reported some degree of language impairment, though 6 participants scored above the conventional WAB-AQ diagnostic cutoff (93.8). Individuals with this type of ‘latent aphasia’ experience communication difficulties and need to concentrate when engaging in language tasks, as well as reduced social participation and difficulties returning to work (Cavanaugh & Haley, 2020). They are included to increase the range and statistical power of the analyses.

The data collection and sharing were approved by Institutional Review Board at the Einstein Healthcare Network; the neuroimaging was conducted at the University of Pennsylvania School of Medicine and approved by its Institutional Review Board. The analyses of de-identified data in the current study were approved by the University of Edinburgh PPLS Research Ethics Committee. We follow recently published reporting guidelines for studies of spoken discourse in aphasia (Stark, Bryant, Themistocleous, den Ouden, & Roberts, 2023; Stark, Dutta, Murray, Bryant, et al., 2021).

### 2.2. Discourse data

#### 2.2.1. CIU

CIU scores were based on 10 samples of connected speech elicited with 10 prompts: two personal questions (‘what you usually do on Sundays’ and ‘where you live and describe it’), two procedural questions (‘how to write and send a letter’ and ‘how to do dishes’), four picture descriptions (‘cat in tree’, ‘birthday cake’; Nicholas & Brookshire, 1993), ‘cookie theft’ (Goodglass, Kaplan, & Barresi, 2001) and ‘picnic scene’ (Kertesz, 2007)), and two picture sequence descriptions (‘farmer and his directions’ and ‘argument’; Nicholas & Brookshire, 1993). During the tasks, participants were encouraged to speak more but were not given any more specific prompts. There was no minimum number of words required, but each participant produced at least 108 words in total. Speech samples were recorded and transcribed by trained technicians. The transcriptions were verbatim and orthographically and phonetically mixed with coders’ annotations using the international phonetic alphabet.

Then transcriptions were coded according to the manual (Nicholas & Brookshire, 1993) and additional disambiguating rules. A word must be intelligible without having to be relevant to the pictures or topics. Nonword fillers were not counted (e.g., uh). Word counts included wrong but intelligible words (e.g., hiscup for hiccup), commentary on the task/speaker (e.g., that is pretty hard), filler words/phrases (e.g., you know), interjections (e.g., oh), informal terms (e.g., nope), and common contractions (e.g., gonna). A CIU was defined as a word that is intelligible, accurate, relevant, and informative to the eliciting stimulus. This included relevant words not used in a grammatically correct manner, mis-production of a word resulting in another word (e.g., paper for pepper), final attempt to correct sound errors, informal terms, and words adding to the events (e.g., he’s going to get hurt). CIUs did not include repetition of ideas, unambiguous pronouns, vague words (e.g., things), conjunctive terms/qualifiers/modifiers used indiscriminately as filler (e.g., ‘Apparently’ this is a kitchen), filler words/phrases, conjunctions (e.g., ‘and’), and commentary.

The CIU coding was done by four trained assistants who achieved more than 94% agreement on words and CIUs on at least 5 participants’ samples. More details are provided in our previous report of these data (Ding et al., 2023). Our prior work found that CIU scores are highly correlated and share similar lesion correlates across elicitation contexts (Ding et al., 2023), so all 10 prompts were used and the CIU measures were averaged across the 10 speech samples for each participant to maximize reliability of the measures. Four standard CIU measures were included in the analysis: #CIU, CIU/min, %CIU and #nonCIU. Previous studies suggest that #CIU and CIU/min are highly correlated with each other, with overall aphasia severity, and lesion size (e.g., Ding et al., 2023). %CIU and #nonCIU may be related to semantic ability (Alyahya, Halai, Conroy, & Lambon Ralph, 2020; Ding et al., 2023; Gordon, 2008; Marchina et al., 2011).

#### 2.2.2. QPA

Participants were asked to retell the story of ‘Cinderella’. If they did not produce more than 150 words, they were asked to retell other well-known stories (e.g., ‘The Little Red Riding Hood’). Retelling the ‘Cinderella’ story is the standard speech sample elicitation task for QPA and, although it is a single prompt, it is more complex and longer (minimum 150 words) than the CIU prompts (minimum 108 words). So the QPA scores are not necessarily based on a smaller sample of speech than the CIU scores are. Their discourse samples were recorded, transcribed and coded by trained technicians following established guidelines (Berndt et al., 2001; Rochon, Saffran, Berndt, & Schwartz, 2000; Saffran et al., 1989). The transcriptions were verbatim and orthographically and phonetically mixed with coders’ annotations using the international phonetic alphabet. An ‘utterance’ was defined by syntax, pause, and intonation (Stark, Dutta, Murray, Fromm, et al., 2021). The coders achieved ~90% agreement on their transcription and coding of utterance boundaries, utterance content, and grammatical structure. More details are provided in our previous report of these data (Zevgolatakou et al., 2023). Standard QPA coding produces many different measures and several studies have examined relationships among them (Ding et al., 2020; Gordon, 2006, 2020; Rochon et al., 2000; Saffran et al., 1989; Zevgolatakou et al., 2023). To align with the number of CIU measures, we also chose four of the most common and reliable QPA measures used in previous analyses of connected speech (Borovsky, Saygin, Bates, & Dronkers, 2007; Bryant, Spencer, & Ferguson, 2017; Halai, Woollams, & Lambon Ralph, 2017; Rezaii, Ren, Quimby, Hochberg, & Dickerson, 2023; Stark, Alexander, et al., 2023): words per minute (WPM: words produced/total speaking duration in minutes), mean sentence length (MSL: words in sentences/sentences produced), inflection index (inflected verbs/inflectable verbs) and auxiliary index ([main verbs + auxiliary elements]/main verbs-1). Theoretically, these measures reflect two different properties of speech: MSL, inflection index, and auxiliary index emphasize the syntactic complexity, while WPM focuses on fluency, a practical and overall measure that includes many processes (Gordon, 2006; Wilson et al., 2010).

### 2.3. Lesion data

Lesion masks were available for 64 participants (47 MRI, 17 CT). For MRI images, lesions were manually drawn by a trained technician on each participant’s T1 image and reviewed by an experienced neurologist. The T1 images were then registered to the Colin27 template by ANTs (Avants, Schoenemann, & Gee, 2006) and their transformation solutions were used to normalize lesion masks. For CT images, lesions were drawn by an experienced neurologist directly onto the Colin27 template based on CT images. See also our previous studies using these lesion masks (Ding et al., 2023; Zevgolatakou et al., 2023).

### 2.4. Statistical analysis

#### 2.4.1. Component and cluster analysis

First, we examined whether participant narrative speech profiles formed clusters based on the speech rate, complexity, and informativeness dimensions. All measures were standardized. Then a principal component analysis (PCA) was applied to the QPA and CIU measures. The number of components was decided through Horn’s parallel analysis using the R package ‘paran’ (https://cran.r-project.org/web/packages/paran/index.html). A varimax rotation was used to extract interpretable components. For the cluster analysis, the cluster number was set to 4 for k-means clustering and was validated by the function ‘fviz_nbclust’ with a method of ‘wss’ from the R package ‘factoextra’ (https://CRAN.R-project.org/package=factoextra). Given the possible difference between discourse elicitation methods (Fergadiotis & Wright, 2011; Stark, 2019), the component and cluster analyses were further separately run on the three discourse genres: complex picture, picture sequence and personal/procedural questions.

#### 2.4.2. Qualitative transcription analysis

In order to get a preliminary, qualitative sense of the different narrative speech patterns, we chose one typical case from each cluster who was matched on one component but varied for the other component compared with the adjacent clusters. The picture description task ‘Cat in tree’ was randomly chosen to examine their speech transcriptions.

#### 2.4.3. Quantitative cluster comparisons

To systematically characterize the properties of different clusters in a quantitative way, we compared multiple behavioral and lesion measures between the clusters. Behavioral measures included CIU measures (#CIU, CIU/min, %CIU, #nonCIU), QPA measures (MSL, WPM, inflection index, auxiliary index), and aphasia severity (WAB-AQ). Clusters were also compared on semantic cognition (Camel and Cactus Test [CCT]) and phonological processing (nonword repetition), but did not show statistically significant differences on these measures (*p* > .07, except for CCT NF-UI vs F–I: *p* = .02); consistent with prior work suggesting that discourse impairments are distinct from semantic and phonological impairments (Zevgolatakou et al., 2023), so they will not be discussed further. Brain measures included lesion percentages of classic language gray matter regions: inferior and middle frontal gyri (IFG, MFG), precentral gyrus (PCG), inferior parietal lobe (IPL), superior and middle temporal gyri (STG, MTG); and white matter tracts: arcuate fasciculus (AF), frontal aslant tract (FAT) (Dick, Garic, Graziano, & Tremblay, 2019; Fedorenko, Hsieh, Nieto-Castañón, Whitfield-Gabrieli, & Kanwisher, 2010; Marchina et al., 2011). The gray matter regions were defined by the Brainnetome atlas (Fan et al., 2016). The white mater tracts were defined by the HCP1065 atlas (Yeh, 2022). The comparisons were done by an ANOVA test among clusters. If the ANOVA result was significant, we further conducted post-hoc pairwise comparisons using the TukeyHSD test.

#### 2.4.4. Lesion classification analysis

The last question was whether the patterns of narrative speech were predictable from lesion patterns. Insofar as they are predictable, neurological damage appears to be (at least partly) responsible for that deficit. Insofar as they are *not* predictable, factors other than the specific lesion pattern appear to be responsible. An alternative factor of particular interest is a communication strategy adjustment by the individual that is not attributable to the specific location of their lesion. We used SVM to classify different clusters by their brain damage patterns. The analysis was run using LIBSVM, with nu-SVC, linear kernel, nu = .3 and other default parameters (Chang & Lin, 2011). *P*-values were based on permutation tests (1000 permutations). Classification was based on a combination of 123 left-hemisphere grey matter regions in the Brainnetome atlas and 34 left-hemisphere and 2 bilateral-hemisphere white matter tracts in the HCP1065 atlas (Fan et al., 2016; Yeh, 2022). Their lesion percentages were calculated by overlapping lesion masks on the regions and then acquiring the proportion of lesion voxels in total region voxels: lesion percentage = Voxels (lesion mask ∩ brain region)/Voxels (brain region). The process was done through a homemade MATLAB script (2018b). Apraxia of speech (AOS) is often present in chronic aphasia (Wilson et al., 2023) and could contribute to fluency deficits (Casilio, Rising, Beeson, Bunton, & Wilson, 2019; Gordon, 2020). Therefore, AOS was added into the model as a covariate to control for motor speech impairment. Four participants’ brain scans were acquired in the (sub-)acute phase (<1 months), when stroke lesions are less stable (Seghier, Ramsden, Lim, Leff, & Price, 2014). Therefore, we validated our results by excluding these lesion masks.

## 3. Results

### 3.1. Component and cluster analysis: four orthogonal clusters

**Fig. 1 –:**
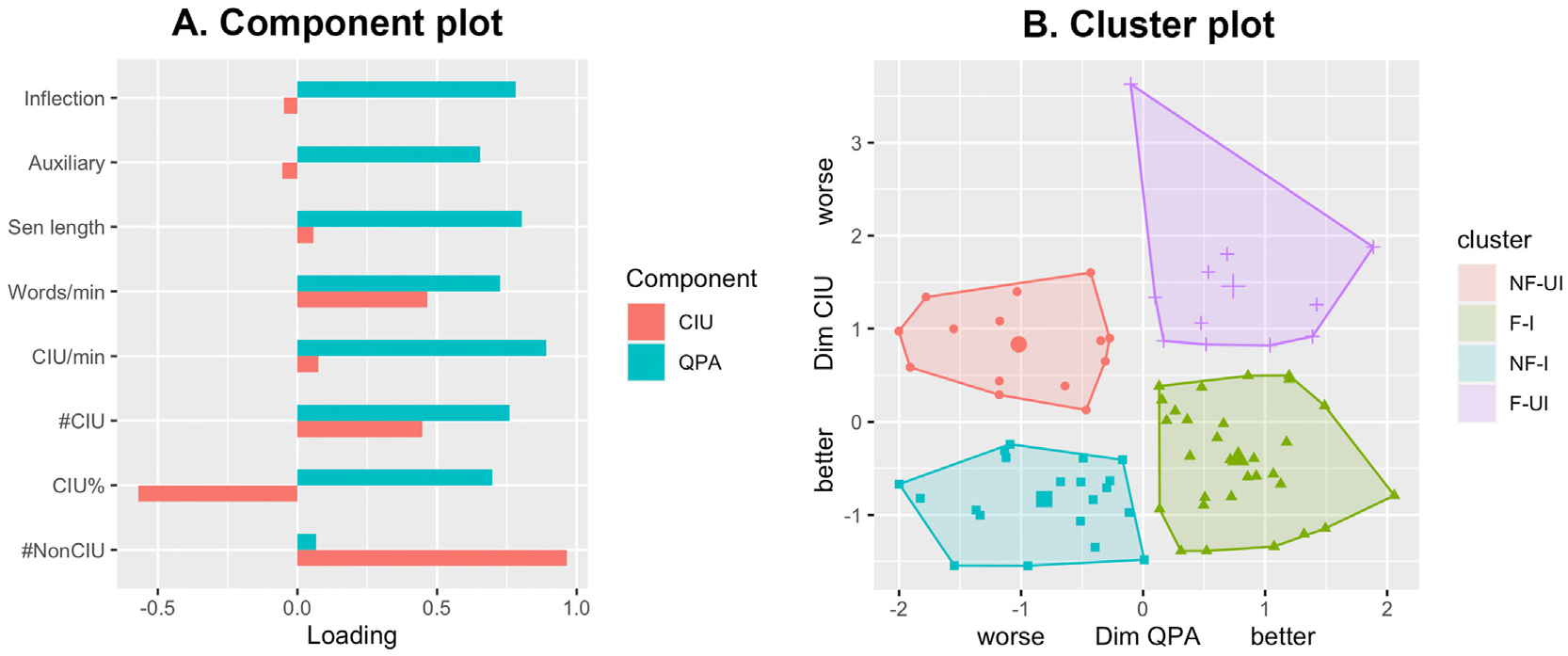
Component and cluster analyses results. (A) Component loadings from the PCA. (B) The four clusters along two dimensions. Higher scores on Dim QPA reflect faster speech rate (words/min) and increased complexity (higher mean sentence length, inflection index, auxiliary index). Higher scores on Dim CIU reflect lower CIU scores (#CIU, CIU/min, %CIU) and more non-CIUs. NF/F: nonfluent/fluent; UI/I: uninformative/informative.

First, the components among all the CIU and QPA measures were extracted (Fig. 1A). Two components were finally extracted according to the Horn’s parallel analysis. The ‘QPA-dominant’ component explained 51% of the variance and had high loadings on all the QPA measures plus #CIU, CIU% and CIU/min (loadings>.65). The ‘CIU-dominant’ component explained 21% of the variance with high loadings on CIU%, #CIU, #NonCIU and Words/min (loadings>.45).

Four orthogonal clusters were separated based on the CIU and QPA measures (Fig. 1B): ‘nonfluent-informative (NF–I; n = 20)’, ‘nonfluent-uninformative (NF-UI; n = 15)’, ‘fluent-informative (F–I; n = 30)’, ‘fluent-uninformative (F-UI; n = 11)’. In total, 70% of the data variance was explained by these clusters. The four clusters were separated by the two independent dimensions: fluency (QPA-dominant dimension) and informativeness (CIU-dominant dimension). ‘Nonfluent’ clusters (shown in red and blue) had more severe impairment on the QPA dimension than ‘fluent’ clusters (shown in green and purple) did. ‘Informative’ clusters (shown in blue and green) performed better on the CIU dimension than ‘uninformative’ clusters did (shown in purple and red).

When the different genres were considered independently, they showed a similar pattern of component and cluster results, except for the oblique cluster result for complex picture description (see Supplementary Figure).

### 3.2. Example cases from each cluster

**Fig. 2 –:**
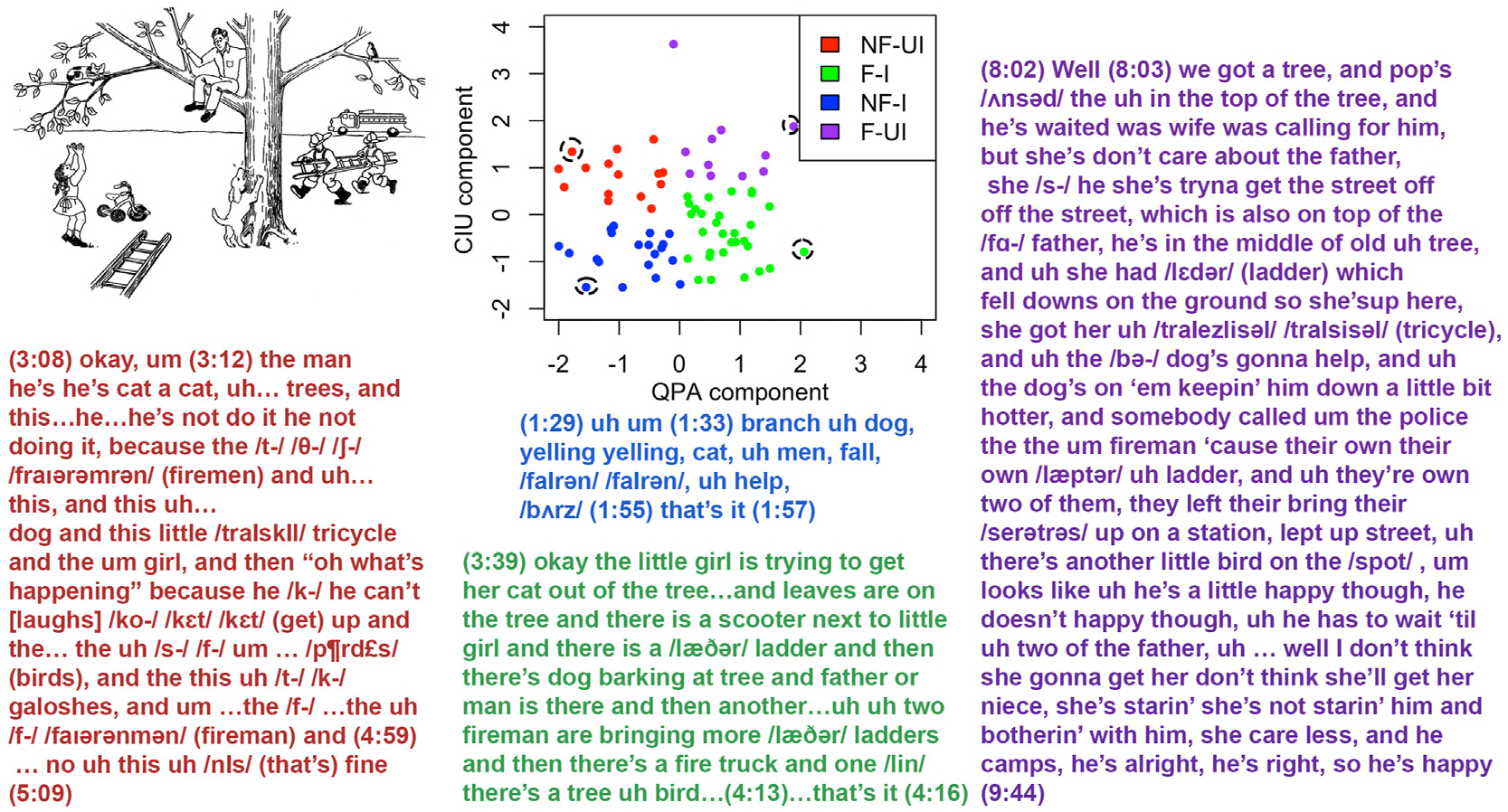
Case transcriptions for different clusters. The scatterplot displays all of the cases color-coded by cluster, with the selected cases from each cluster highlighted. The text shows transcriptions of the picture descriptions for ‘cat in tree’ (top left). CIU component scores are flipped for a better understanding. NF/F: nonfluent/fluent; UI/I: uninformative/informative.

After the clusters were identified, we began to characterize their speech properties by looking at some typical cases’ transcriptions. Four matched cases, one from each cluster, were picked based on %CIU and speech rate (WPM) such that they differed on one dimension while being approximately matched on the other (Fig. 2). Cases from the two ‘nonfluent’ clusters (shown in red and blue) and the two ‘fluent’ clusters (shown in green and purple) had similar fluency (QPA-dominant component score) but different informativeness (CIU-dominant component score); cases from the two ‘informative’ clusters (shown in green and blue) and the two ‘uninformative’ clusters (shown in red and purple) had similar informativeness (CIU-dominant component score) but different fluency (QPA-dominant component score).

The transcriptions from the four cases are shown in Fig. 2. The case from the ‘NF–I’ cluster (shown in blue) produced very few words, which were almost always isolated nouns or verbs rather than sentences or phrases, and stopped after about 30 s. The case from the ‘NF-UI’ cluster (shown in red) spoke for about 2 min and produced many more words, but there were many hesitations (filled and unfilled pauses) and substantial phonological struggle. As a result, the number of words per minute was still fairly low, as was the proportion of informative words (%CIU). The cases from the two ‘fluent’ clusters both spoke relatively fluently in the sense of producing words at a rate similar to unimpaired speakers (neurologically healthy speech rates are typically 125–200 words per minute; e.g., Liberman, 2010; Yuan, Liberman, & Cieri, 2006). The case from the ‘F–I’ cluster (shown in green) mostly produced informative words with only a few errors – close to the speech pattern that would be expected from an unimpaired speaker. The case from the ‘F-UI’ cluster (shown in purple) produced relatively well-structured sentences, or at least sensible multi-word phrases, but with many circumlocutions, resulting in a relatively low proportion of informative words (%CIU).

### 3.3. Comprehensive comparison among four clusters

**Fig. 3 –:**
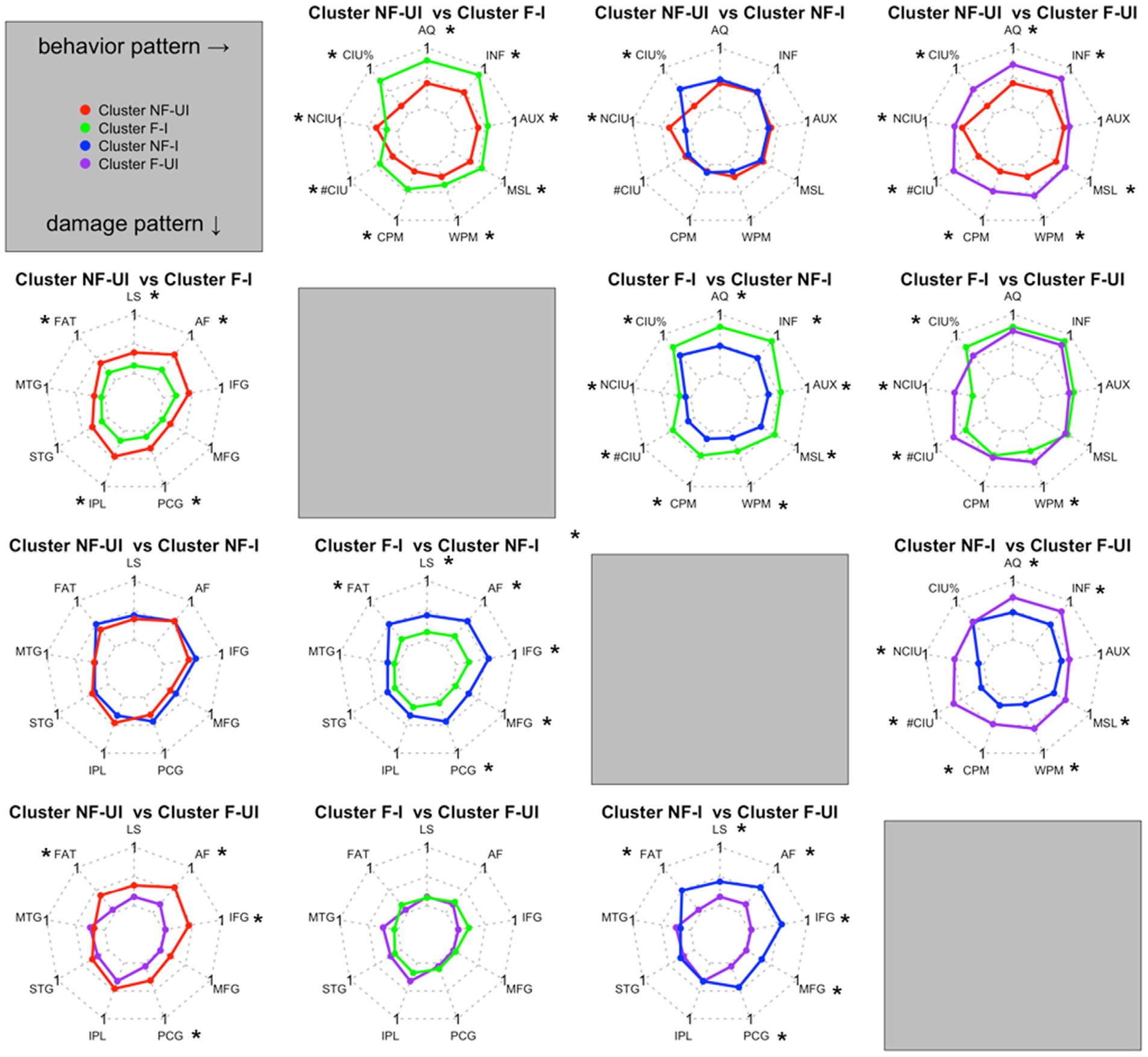
Radar maps for comparisons of behavioral and lesion measures. * indicates *p* < .05 after post-hoc tests. All the measures were scaled from 0 to 1 to be comparable between each other. The upper triangle shows comparisons on behavioral measures; the lower triangle shows comparisons on lesion measures. NF/F: nonfluent/fluent; UI/I: uninformative/informative; AQ: aphasia quotient; INF: inflection index; AUX: auxiliary index; MSL: mean sentence length; WPM: words per minute; CPM: CIU per minute; NCIU: non-CIU; LS: lesion size; AF: arcuate fasciculus; FAT: frontal aslant tract; IFG: inferior frontal gyrus; MFG: middle frontal gyrus; PCG: precentral gyrus; IPL: inferior parietal lobe; STG: superior temporal gyrus; MTG: middle temporal gyrus.

Next, we comprehensively compared behavioral and brain damage differences between clusters (Fig. 3). The two ‘fluent’ clusters (shown in green and purple) produced longer sentences, at a faster rate, with more information (#CIU) and more complex syntax (inflection index) than the two ‘non-fluent’ clusters (shown in red and blue) did (*p* < .01). They also had overall milder aphasia (higher WAB-AQ; *p* < .002). Lesion patterns were also significantly different between the two ‘fluent’ clusters compared to the two ‘nonfluent’ clusters. The NF–I cluster (shown in blue) had larger total lesions than the fluent clusters (green and purple) and more damage in the AF and FAT and frontal grey matter (IFG, MFG, PCG) (*p* < .004). The NF-UI cluster (shown in red) was less distinct from the fluent clusters in terms of lesion patterns: their lesions were not consistently statistically significantly larger though they had more damage to AF, FAT and PCG (*p* < .03).

Within ‘fluent’ and ‘nonfluent’ cluster pairs, the clusters had similar speech rate and complexity, but differed in informativeness. The ‘NF–I’ cluster (shown in blue) produced fewer non-CIUs and higher %CIU than the NF-UI cluster (shown in red) did (*p* < .0001). The ‘F–I’ cluster (shown in green) also produced fewer non-CIU and higher %CIU than the ‘F-UI’ cluster (shown in purple) did, together with more CIUs and words per minute (*p* < .003). There were no statistically significant differences in lesion measures between informativeness clusters within fluency clusters (i.e., NF–I vs NF-UI, F–I vs F-UI; *p* > .20).

### 3.4. ROI-based classification

**Fig. 4 –:**
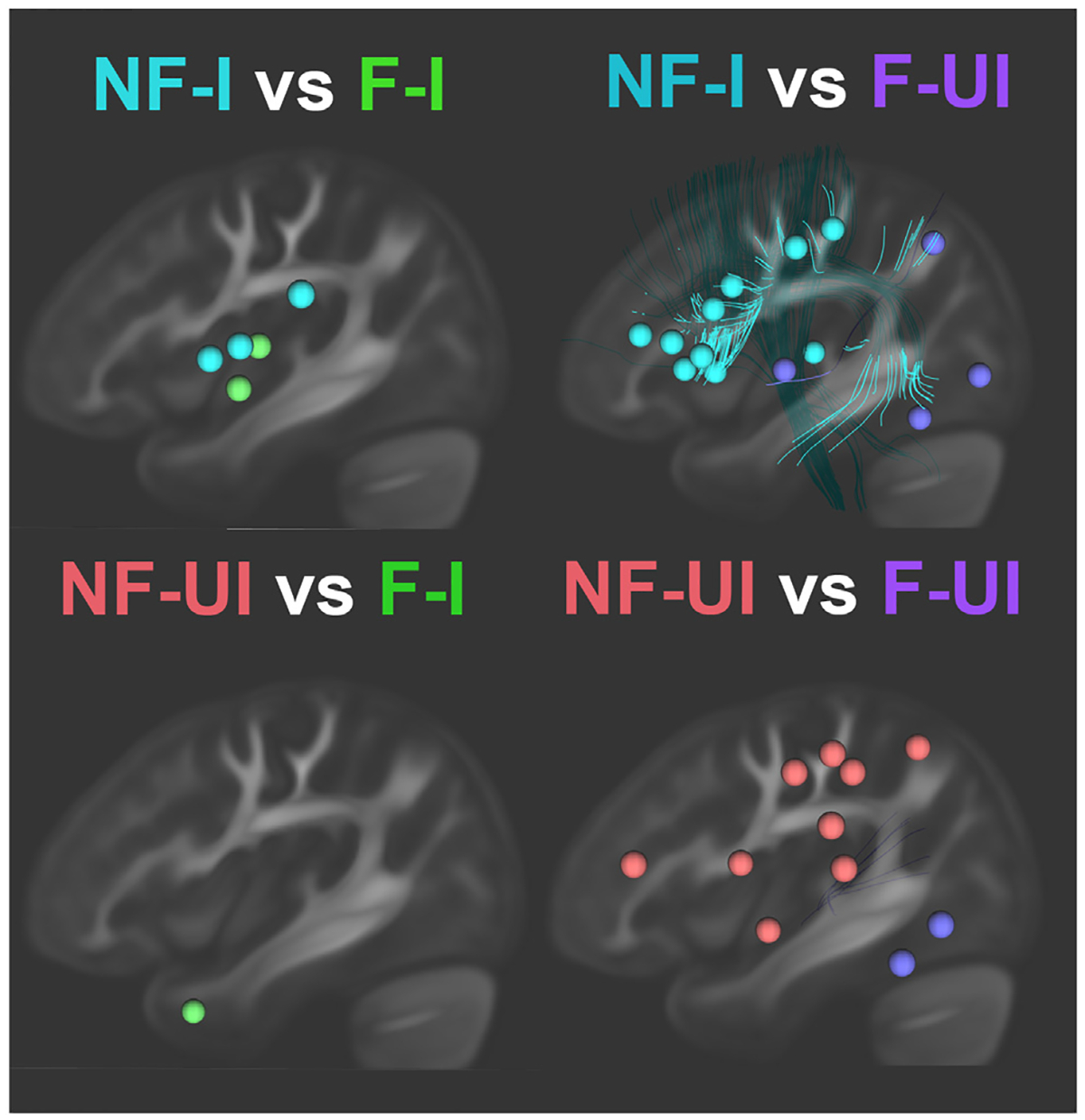
The influential GM/WM regions in the model with scaled weights >5. The color of regions indicates participants in that cluster have more damage on those regions. Specific region names are listed in Table 2. NF/F: nonfluent/fluent; UI/I: uninformative/informative.

To further evaluate whether the clusters can be distinguished based on lesion patters, we used SVM models trained on GM and WM ROI damage to classify the 4 clusters (Table 2). The model successfully distinguished ‘Fluent’ clusters from ‘Nonfluent’ clusters (>67%, *p* < .05). Frontoparietal regions and dorsal white matter tracts, together with AOS, contributed most strongly (highest weights) to ‘Nonfluent’ clusters (Fig. 4; shown in red and blue). The model was not able to significantly distinguish ‘Informative’ clusters from ‘Uninformative’ clusters (<68%, *p* > .23). When the four acute lesion masks were excluded, the results did not change substantially (see Supplementary Table).

## 4. Discussion

We combined measures of the rate, complexity, and informativeness of narrative speech to classify individuals with post-stroke aphasia and tested cognitive and neurological contributions to the classification. We found that participants could be classified into four orthogonal clusters based on two dimensions: (1) a QPA-dominant component reflecting speech rate and complexity, and (2) a CIU-dominant component reflecting discourse informativeness. Individuals with higher speech rate and complexity had less severe aphasia, smaller lesions, and clusters along this dimension could be distinguished based on lesion patterns. In contrast, differences in informativeness were less clearly associated with cognitive or neural profiles, suggesting that they may be a result of strategic choices rather than cognitive or neurological deficits. The following sections discuss these results and our interpretation in more detail, along with potential applications of these findings.

### 4.1. Two dimensions, four clusters

QPA and CIU were developed to capture very different aspects of narrative speech in aphasia: structural and functional, respectively (Armstrong, 2000; Nicholas & Brookshire, 1993; Saffran et al., 1989). Both approaches produce numerous measures that are associated with different theoretical claims, though these are not always distinguishable in practice (Ding et al., 2020; Gordon, 2006, 2008, 2020; Zevgolatakou et al., 2023). For this study, we selected 4 QPA measures and 4 CIU measures that appeared to capture distinct aspects of narrative speech. Words per minute was intended to capture basic speech rate, which itself relies on many cognitive processes, such as motor and syntax processes (Casilio et al., 2019; Gordon, 2020; Mirman et al., 2019). MSL, inflection index, and auxiliary index capture structural complexity and are thought to reflect syntactic processing in connected speech of individuals with aphasia (Fromm, Greenhouse, Pudil, Shi, & MacWhinney, 2022; Gleichgerrcht et al., 2021; Thompson et al., 2013). CIU measures – #CIUs, CIU/min, %CIU, and #non-CIUs – capture discourse informativeness with somewhat different points of emphasis (quantity, speed, efficiency). These measures are thought to reflect lexical-semantic processes in discourse contexts and are used to test whether word retrieval therapy (e.g., semantic feature analysis; naming cueing) generalizes to discourse, though the results are often disappointing (e.g., Cavanaugh et al., 2024).

Principal components analysis was applied to these measures in order to capture distinct aspects of narrative speech. The component scores were then used to examine what cognitive and neurological factors are associated with differences in these distinct aspects of narrative speech. That we found distinct QPA-based and CIU-based dimensions is not, in itself, surprising. QPA and CIU measures are based on different principles, and are derived from somewhat different speech tasks. Consistent with previous studies that combined micro- and macro-structural measures, QPA measures (including rate and complexity), formed a single dimension that reflects features of agrammatic aphasia (Faroqi-Shah, 2023; Gordon, 2020; Halai et al., 2017; Saffran et al., 1989; Zevgolatakou et al., 2023). Fewer studies have conducted component analyses of CIU measures. We found that CIU/min was strongly associated with QPA measures, suggesting that it is strongly influenced by speech rate. In contrast, %CIU, #CIU and #nonCIU were more distinct from QPA measures, suggesting these measures are more sensitive to informativeness in narrative speech of aphasia. However, the measures’ contributions to the two components are not absolutely separated. Quantity and speed of relevant information (#CIU and CIU/min) were associated with words per minute and other QPA measures, consistent with previous findings (Alyahya et al., 2020; Wang, Marchina, Norton, Wan, & Schlaug, 2013), and indicating (not surprisingly) that difficulty producing relevant information is closely related to impairments of speech rate and complexity. Non-CIU production emerged as the most informativeness-specific measure (i.e., loading strongly on the CIU component but not on the QPA component), suggesting that willingness to produce non-informative content may be a separate dimension from traditional considerations of fluency.

Availability of larger data sets that include neuropsychological tests and lesion patterns and of sophisticated clustering algorithms have allowed researchers to explore different aphasia classification schemes (Butler, Lambon Ralph, & Woollams, 2014; Corbetta et al., 2015; Landrigan et al., 2021; Zhao, Halai, & Lambon Ralph, 2020). These data-driven studies highlight the importance of different deficit dimensions or distinctions, such as phonological vs semantic (Landrigan et al., 2021) or total number of words vs number of closed-class words in connected speech (Fromm et al., 2022). The present study adds to these efforts by contrasting complexity/fluency and informativeness dimensions and demonstrating that the complexity/fluency dimension is closely related to cognitive and neurological profiles whereas the informativeness dimension is less so.

We make the further claim that when differences in narrative speech are not attributable to cognitive or neurological differences, they may be a result of differences in communication strategy. For example, producing isolated highly informative words while sacrificing syntactic structure (high CIU scores but low sentence length and complexity) or trying to maintain a fluent speech rate but producing circumlocutions, irrelevant words, or phonological/articulatory errors in the process (high WPM but low CIU%).

### 4.2. Severity and lesion pattern differences between ‘fluent’ and ‘nonfluent’ clusters

The two ‘fluent’ clusters differed from the two ‘nonfluent’ clusters on QPA-based measures of speech rate (WPM) and complexity (MSL, inflection index, and auxiliary index). These differences were also associated with differences in aphasia severity (WAB-AQ) and total lesion size, indicating that these fluency/structural impairments are closely related to general severity (Gordon, 2006; Zevgolatakou et al., 2023).

Our SVM results revealed that the distinction between these pairs of clusters was also predictable from lesion patterns. Speech rate and complexity differences were highly predictable from damage to frontoparietal grey matter regions and dorsal white matter pathways connecting these regions. This is consistent with conclusions from previous LSM studies (Basilakos et al., 2014; Ding et al., 2020; Zevgolatakou et al., 2023). Apraxia of speech is common in chronic aphasia (Clough & Gordon, 2020; Wilson et al., 2023), and this factor also helps to distinguish ‘nonfluent’ and ‘fluent’ clusters, consistent with its role in non-fluent output (Casilio et al., 2019; Gordon, 2020; Matchin et al., 2020). However, the lesion correlates and predictors remain after controlling for AOS, indicating that the ‘fluency’ dimension captures higher-level language deficits in addition to articulatory-motor issues.

### 4.3. Communication strategy differences between ‘uninformative’ and ‘informative’ clusters

The difference between ‘uninformative’ clusters and ‘informative’ clusters corresponded to differences in the CIU-dominant component of narrative speech (particularly #CIU, CIU%, and non-CIUs). Unlike the rate/complexity difference, this difference was not associated with other language-cognitive differences: these clusters were approximately equal on QPA measures and WAB AQ (as well as phonological and single word processing measures). They were also not distinguished by total lesion size or damage to particular regions within the language network. SVM analysis further showed that patterns of brain damage were not very effective at predicting these cluster differences, especially the difference between the more severely impaired ‘NF-UI’ and ‘NF–I’ clusters (i.e., informativeness of speech among nonfluent individuals). Previous studies have found that #CIU and CIU/min were associated with damage to dorsal white matter tracts (Ding et al., 2023; Keser, Meier, Stockbridge, & Hillis, 2020; Marchina et al., 2011). The lack of association in the present study may result from inclusion of #non-CIU and %CIU, which reflect efficiency of information rather than quantity and speed. These clusters were also not distinguished by apraxia of speech, which was a significant difference between ‘fluent’ and ‘non-fluent’ clusters, indicating that speech informativeness is not strongly affected by motor-articulatory impairments.

The lack of cognitive-linguistic or lesion correlates for this difference suggests that it might reflect a strategic choice. ‘NF-UI’ and ‘F–I’ clusters differed substantially in terms of speech rate and sentence length, but individuals in both of those clusters produced relatively normal syntax. In contrast, individuals in ‘NF–I’ cluster had similar speech rate to those in ‘NF-UI’ cluster, but restricted their output to single informative words. Individuals in ‘F-UI’ cluster had similar speech rate to those in ‘F–I’ cluster, but this came at the price of many irrelevant words.

### 4.4. Limitations

There are several limitations for this study. First, previous studies found differences across discourse elicitation methods (Fergadiotis & Wright, 2011; Stark, 2019). Our validation results found a very consistent pattern, but complex picture description produced a slightly different pattern of clusters compared to the other two genres: #CIU was more associated with the QPA component and there were three clusters across the QPA dimension. CIU measures may be less sensitive in complex picture description because participants can use visual cues to retrieve relevant/informative words and because the single picture is more static than other elicitation genres (which typically require temporal and spatial shifts between events). As a result, generating content in this genre is easier and content diversity is lower (Alyahya et al., 2020; Fergadiotis & Wright, 2011; Schnur & Wang, 2024), possibly allowing participants to focus more on syntactic processing (Stark, 2019). Although this is a relatively small deviation against a background of high consistency across elicitation genres, future research should consider the impact of elicitation type during study design and when interpreting results. More generally, CIU and QPA are calculated based on different elicitation methods (QPA: longer-form storytelling; CIU: procedural/personal information, complex picture description, picture sequence descriptions). Such a methodological mismatch can also be considered as a confounding factor. Future studies could use the same speech samples to study the relationship between QPA and CIU measures.

We propose strategic differences to explain the absence of a clear cognitive and neurological distinction between informative and uninformative clusters, but other explanations are also possible. For example, other important cognitive factors, such as working memory are not included in the current study (Martin & Schnur, 2019). Also, informativeness may rely on a distributed set of brain regions or reflect multiple deficits, each with their own neural correlate. We used a multivariate method (linear-kernel SVM) for lesion-based classification, which should be sensitive to distinct region contributions, but that does not guarantee that it will detect any possible set of lesion correlates. Further research could use other methods to further investigate this. For example, changing to a non-linear kernel for SVM, optimizing SVM parameters through grid-search, or using other algorithms (e.g., random forest, ridge regression, sparse canonical correlation; Corbetta et al., 2015; Pustina et al., 2017, 2018).

### 4.5. Implications and future directions

“Fluency” is a foundational and central construct in aphasia classification, but it is broadly agreed that many different cognitive and neural sub-systems contribute to fluent speech production. The present study contributes to ongoing efforts to identify the core dimensions or components of fluency and their neural correlates. Here we show that speech rate and complexity are closely related to aphasia severity and lesion size, and more specifically to frontoparietal and dorsal white matter damage, which converges with results from our research group and others (Ding et al., 2020; Gordon, 2008; Halai et al., 2017; Wilson et al., 2010; Zevgolatakou et al., 2023). In contrast, speech informativeness, as measured by CIU measures, appears to reflect strategic differences rather than cognitive or neurological impairments. These distinctions may help to better characterize narrative speech deficits and to develop individualized treatment plans.

Other recent studies also suggest that individuals with aphasia make strategic choices about their communication patterns (Fedorenko et al., 2023; Gordon, 2008; Rezaii, Michaelov, et al., 2023). We do not claim that all strategic choices are necessarily under conscious control. They may be influenced by other factors, such as age, personality factors, or long-standing speaking habits. We also do not know whether the communication strategies identified in this study are limited to controlled speech elicitation tasks or reflect the participants’ real-world speech patterns, nor do we know how these strategies contribute to communication success. These are important questions for future research.

A potential implication of such research would be treatment strategies that aim to shift communication strategies toward more effective ones. These could be meta-cognitive or life participation approaches (Chapey et al., 2018; Kersey et al., 2021), or treatments designed to use spared abilities to compensate for impaired abilities (Fridriksson & Hillis, 2021; Suárez-González et al., 2021). Another approach would be to use what we have learned about the lesion correlates – such as that left frontoparietal grey matter and dorsal white matter damage are associated with impaired speech rate and complexity – to inform brain stimulation or pharmacological treatment approaches (Bucur & Papagno, 2019; Wan, Zheng, Marchina, Norton, & Schlaug, 2014).

## 5. Conclusion

In conclusion, the present study distinguished rate/complexity and informativeness dimensions of narrative speech in post-stroke aphasia. The rate/complexity dimension was related to aphasia and lesion severity, especially damage to frontoparietal regions. In contrast, the informativeness dimension was not clearly associated with cognitive-linguistic or neurological dimensions and may reflect choices about communication strategy, especially for more severely impaired individuals.

## Supplementary Material

1

Supplementary data

Supplementary data to this article can be found online at https://doi.org/10.1016/j.cortex.2025.03.006.

## Figures and Tables

**Table 1 –:** Demographic and neuropsychological information.

Variable	Num or Mean, Median	IQR	Range
Sex (Female:Male)	Num = 35:41		
Race (African-American:Caucasian)	Num = 36:40		
Age (years)	M = 58, Med = 58	50–68	30–79
Education (years)	M = 15, Med = 14	12–18	10–21
Months post onset	M = 54, Med = 24	9–81	4–270
Total lesion size (cc)	M = 100, Med = 77	46–138	5–376
WAB AQ (max 100)	M = 79, Med = 81	71–90	47–99
Aphasia type (Anomic: Broca: Conduction: Transcortical sensory; Transcortical motor: Wernicke)	Num = 43:8:10:2:4:2		
Apraxia of speech (1–4)	Num = 50:7:8:4		
Nonword repetition (% correct)	M = 51%, Med = 52%	32%–70%	0%–95%
Camel and cactus test (% correct)	M = 76%, Med = 79%	72–83	31–94
Philadelphia naming test (% correct)	M = 74%, Med = 79%	64%–86%	15%–97%
Number of CIUs	M = 56, Med = 51	28–73	6–161
Number of Non-CIUs	M = 42, Med = 37	21–60	3–163
%CIUs	M = 58%, Med = 60%	49%–70%	25%–86%
CIUs per minute	M = 76, Med = 72	50–97	15–195
Mean sentence length (QPA; words)	M = 6, Med = 6	5–7	2–12
Words per minute (QPA)	M = 68, Med = 62	42–85	14–194
Inflection index (QPA)	M = .67 Med = .75	.57–.89	0–1
Auxiliary complex index (QPA)	M = 1, Med = 1	.79–1.26	0–2.23

WAB: Western Aphasia Battery; AQ: aphasia quotient; CIU: correct information units; QPA: quantitative production analysis; Camel and cactus test contains 64 picture matching trials (Bozeat, Lambon Ralph, Patterson, Garrard, & Hodges, 2000).

**Table 2 –:** Cluster classification results.

Cluster contrast	Accuracy	AOS	Regions
NF–I vs F–I	**77%, *P*** < **.001**	NF–I	GM (NF–I): IPL6, INS5/6 GM (F–I): BG3, THA8
NF-UI vs F-UI	**67%, *P*** = **.05**	NF-UI	GM (NF-UI): IFG2, STG2, SPL5, IPL3/6, PoG1/3, BG3/5 GM (F-UI): ITG5/6 WM(F-UI): AR
NF–I vs F-UI	**93%, *P*** < **.001**	NF–I	GM (NF–I): IFG1/2/3/5/6, PrG6, PoG1/3, INS3, THA3 WM (NF–I): AF, CBT, CPT_F, CPT_P, CST, CS_S, DRTT, FAT, ML, RST, SLF2, TR_S GM(F-UI): STG3, ITG2, IPL2, LOC2 WM(F-UI): C_PHP, MdLF
NF-UI vs F–I	**76%, *P*** = **.02**	NF-UI	GM(F–I): STG1
NF–I vs NF-UI	50%, *p* = .54		
F–I vs F-UI	68%, *p* = .23		

First column identifies the cluster comparison, second column shows the classification accuracy with permutation-based *p*-value, third column displays which cluster membership AOS (apraxia of speech) is positively associated with, last column lists the regions where damage is positively associated with cluster membership. Statistically significant effects are shown in bold. The numbers following grey matter regions are labels from the Brainconnectome Atlas. NF/F: nonfluent/fluent; UI/I: uninformative/informative; GM: gray matter; IPL/SPL: inferior/superior parietal lobe; INS: insula; BG: basal ganglia; THA: thalamus; IFG: inferior frontal gyrus; STG/ITG: superior/inferior temporal gyrus; PoG/PrG: post/pre-central gyrus; LOC: lateral occipital cortex; WM: white matter; AR: acoustic radiation; AF: arcuate fasciculus; CBT: corticobulbar tract; CPT_F/P: frontal/parietal part of corticopontine tract; CST: corticospinal tract; CS_S: superior corticostriatal tract; DRTT: dentatorubrothalamic tract; FAT: frontal aslant tract; ML: medial lemniscus; RST: reticulospinal tract; SLF: superior longitudinal fasciculus; TR_S: superior thalamic radiation; C_PHP: parahippocampal and parietal part of cingulum; MdLF: middle longitudinal fasciculus.

## Data Availability

DATA: Some raw and processed data supporting this research are publicly available, while some are subject to restrictions: https://osf.io/pk7e9/ CODE: All analysis code supporting this research is publicly available: https://osf.io/pk7e9/ MATERIALS: This research did not make use of any materials to generate or acquire data. DESIGN: This article reports, for all studies, how the author(s) determined all sample sizes, all data exclusions, all data inclusion and exclusion criteria, and whether inclusion and exclusion criteria were established prior to data analysis. PRE-REGISTRATION: No part of the study procedures was pre-registered in a time-stamped, institutional registry prior to the research being conducted. No part of the analysis plans was pre-registered in a time-stamped, institutional registry prior to the research being conducted.
