# Supplementary material for: Data-driven classification of narrative speech characteristics in stroke aphasia distinguishes neurological and strategic contributions
Source: Cortex. Author manuscript; Available in PMC 2026 May 18. (PMC13181383; doi:10.1016/j.cortex.2025.03.006)
Supplement: 1 [file NIHMS2172873-supplement-1.docx]

**
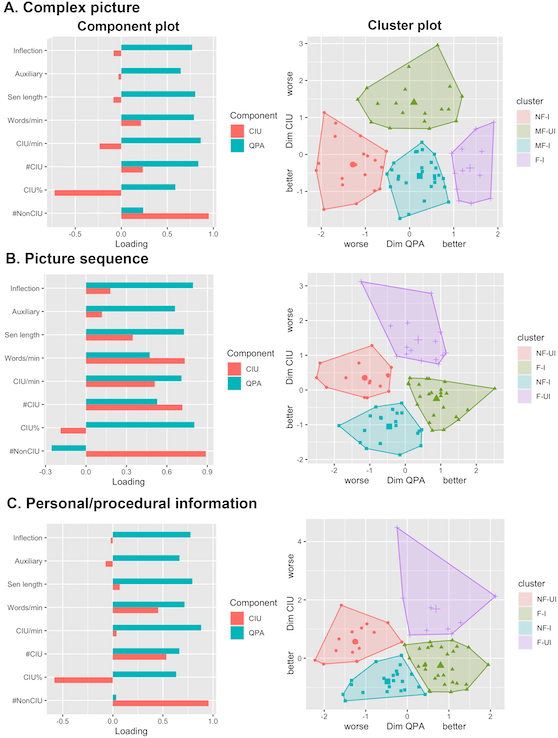
**

**Supplementary Figure.** Component and cluster analyses results of different elicitation methods. The left column shows component loadings of the component analysis. The right column shows 4 clusters along 2 dimensions. CIU: correct information unit; QPA: quantitative production analysis; NF/MF/F: nonfluent/medium fluent/fluent; UI/I: uninformative/informative.

**Supplementary Table. Cluster classification results with lesion masks more than 1 month**

| Cluster contrast | Accuracy | AOS | Regions |
| --- | --- | --- | --- |
| NF-I vs F-I | **76%, p<0.001** | NF-I | GM (NF-I): IPL6, INS5/6  GM (F-I): BG3 |
| NF-UI vs F-UI | **74%, p=0.02** | NF-UI | GM (NF-UI): IFG1, PrG6, STG2, SPL3/5, IPL2/3/4, PoG3, BG5  WM(NF-UI): SLF2/3 |
| NF-I vs F-UI | **96%, p<0.001** | NF-I | GM (NF-I): MFG2, IFG1/3/5/6, PrG6, IPL3, PoG1/3, INS3/6  WM (NF-I): AF, CBT, CPT_F, CPT_P, CST, CS_S, DRTT, EMC, FAT, ML, TR_S  GM(F-UI): PCun3  WM(F-UI): C_PHP |
| NF-UI vs F-I | **0.77%, p=0.01** | NF-UI | GM(F-I): STG1 |
| NF-I vs NF-UI | 0.50%, p=0.54 |  |  |
| F-I vs F-UI | 73%, p=0.10 |  |  |

First column identifies the cluster comparison, second column shows the classification accuracy with permutation-based p-value, third column displays which cluster membership AOS (apraxia of speech) is positively associated with, last column lists the regions where damage is positively associated with cluster membership. Statistically significant effects are shown in bold. The numbers following gray matter regions are labels from the Brainconnectome Atlas. NF/F: nonfluent/fluent; UI/I: uninformative/informative; GM: gray matter; IPL/SPL: inferior/superior parietal lobe; INS: insula; BG: basal ganglia; MFG/IFG: middle/inferior frontal gyrus; STG: superior temporal gyrus; PoG/PrG: post/pre-central gyrus; WM: white matter; AF: arcuate fasciculus; CBT: corticobulbar tract; CPT_F/P: frontal/parietal part of corticopontine tract; CST: corticospinal tract; CS_S: superior corticostriatal tract; DRTT: dentatorubrothalamic tract; EMC: extreme capsule; FAT: frontal aslant tract; ML: medial lemniscus; SLF: superior longitudinal fasciculus; TR_S: superior thalamic radiation; C_PHP: parahippocampal and parietal part of cingulum.
